# Is Pectus Excavatum a Risk Factor for Spontaneous Pneumothorax? “Haller Index Measurements in Patients with Primary Spontaneous Pneumothorax”

**DOI:** 10.1155/2019/3291628

**Published:** 2019-04-02

**Authors:** Ali Kılıçgün, Osman Yakşi, Mehmet Ünal

**Affiliations:** Department of Thoracic Surgery, Abant Izzet Baysal University, Bolu, Turkey

## Abstract

**Aim:**

In this study, we aimed to retrospectively investigate whether pectus excavatum (PE) is a risk factor for the development of primary spontaneous pneumothorax (PSP) and to determine its role in the etiology of the disease.

**Materials and Methods:**

Chest-computed tomography (CT) of the patients who were treated for spontaneous pneumothorax between January 2015 and December 2017 in our clinic was examined, and their Haller indices were measured (group I). The patients in the control group who underwent chest CT for other reasons during the same period and were in the same age with the group I were also included in the study (group II) *Results.* In group I, for patients with PE, the mean Haller index was 2.41, while it was 2.09 in the control group (group II). There was a significant difference between the two groups.

**Conclusions:**

PE deformities in the chest wall may predispose to the development of spontaneous pneumothorax, and PE may be among the etiologic factors of spontaneous pneumothorax. Therefore, there is a need for studies involving larger patient groups.

## 1. Introduction

Pectus excavatum (PE) is the most common chest deformity. The incidence is about 1 in 300–400 live births. It is 4 times more common in males [1]. The Haller index is used to measure the depth of the chest deformity and to decide the operation. Haller et al. described this index in 1987. According to this description, the Haller index is the ratio of the transverse diameter to the anterior-posterior diameter in the deepest portion of the deformity in the chest CT. The Haller index below 2 is accepted as normal. It was emphasized that surgery could be recommended when this ratio is above 3.25 [2]. Pneumothorax is the accumulation of air in the pleural space. Spontaneous pneumothorax is of two categories: primary and secondary. The primary spontaneous pneumothorax (PSP) is usually due to the rupture of the subpleural blebs, while the etiology of the secondary spontaneous pneumothorax includes various lung diseases. There are studies investigating the (abnormalities) disorders of bone and cartilage metabolism in PE and PSP. However, no common reason was found for both of the diseases [3, 4]. Besides, genetic studies could not reveal the pathogenesis of both PE and PSP [5].

However, chest wall deformities, PE in particular, are not mentioned in the textbooks [6–8]. We investigated that whether PE is a risk factor for the development of spontaneous pneumothorax.

## 2. Materials and Methods

**Figure 1:**
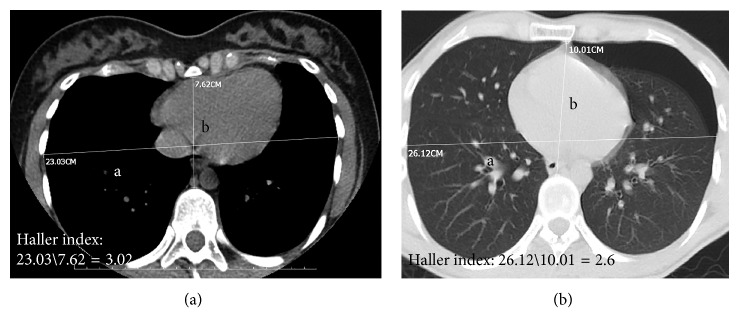
Measurement of the Haller index in (a) mediastinal section of thorax tomography and (b) parenchymal section of thorax tomography. Pneumothorax was seen on the left.

Twenty patients who were treated for spontaneous pneumothorax and underwent chest CT between January 2015 and December 2017 were included in our study (group I: pneumothorax group). The control group (group II) included patients who underwent chest CT for other reasons during the same period and were in the same age range as group I (20–35 years). Both groups included 20 patients with complete hospital records. Exclusion criteria are indicated in Table 1. The diagnoses of the patients in the control group are shown in Table 2. Age, gender, posteroanterior and lateral chest X-ray, complete blood count and biochemical results, additional diseases, smoking, treatment, and operations were noted. Group I included patients with primary spontaneous pneumothorax, and those with secondary pneumothorax were not included in the study. The Haller indices of the patients in both groups were measured (Figures 1(a) and 1(b)). The Haller indices of the two groups were compared with the Mann–Whitney *U* test.

## 3. Results

### 3.1. Group I

Pneumothorax group consisted of 20 patients with primary spontaneous pneumothorax. The mean age was 25.4 (20–35 years). Seven patients smoked (35%) while 13 did not. Two patients received oxygen therapy, and 18 received tube thoracostomy and underwater sealed drainage therapy. Three patients (15%) underwent video-assisted thoracic surgery (VATS) due to prolonged air leak.

### 3.2. Group II

Control group consisted of 20 patients who underwent chest CT during the same period for reasons other than spontaneous pneumothorax. The mean age was 26.2 (20–35 years). Five patients smoked (25%) while 15 did not.

**Figure 2:**
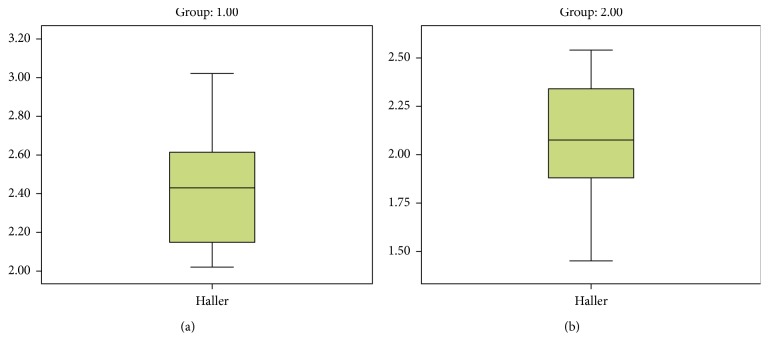
(a) Group I graph of the Haller index in patients with spontaneous pneumothorax. (b) Group II graph of the Haller index in the control group.

**Figure 3:**
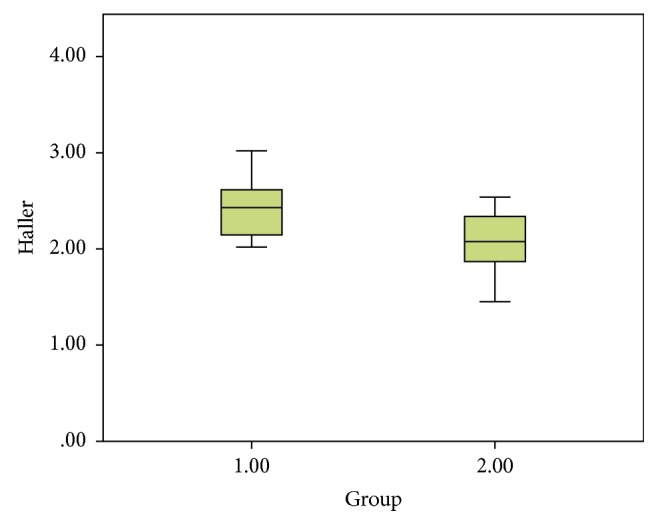
Statistical graph comparing the group with spontaneous pneumothorax (group I) and the control group (group II).

There was no difference between the age distributions of the two groups. Biochemical analysis and complete blood count were normal in both PE and PSP patients when the Haller indices were 2.41 in patients with PE (group I) (Figure 2(a)) and 2.09 in the control group (group II) (Figure 2(b)). There was a significant difference between the two groups (*p*=0.006) (Figure 3).

## 4. Discussion

Pectus excavatum (PE) is the most common chest deformity. The incidence is about 1 in 300–400 live births. It is 4 times more common in males [1]. The Haller index is used to measure the depth of the chest deformity and to decide the operation. Haller et al. described this index in 1987. It was emphasized that surgery could be recommended when this ratio is above 3.25. In the same study, it was noted that this index could be used for screening in children and adolescents [2]. However, recent studies have shown that subjective complaints in patients with PE are not related to the Haller index; for example, these complaints are independent of the size of the deformity [6]. In patients with severe PE, preoperative evaluation showed reduced pulmonary function by developing restrictive pulmonary pattern [7].

A majority of patients have mild PE, have no symptoms, and do not need treatment. However in our study, we investigated whether spontaneous pneumothorax could develop more frequently in these patients.

Pneumothorax is the accumulation of air in the pleural space. Spontaneous pneumothorax is of two categories: primary and secondary. The primary spontaneous pneumothorax is usually due to the rupture of the subpleural blebs, while the etiology of the secondary spontaneous pneumothorax includes various lung diseases.

However, chest wall deformities, PE in particular, are not mentioned in the textbooks [8]. Our study suggests that PE slightly increases the risk of developing spontaneous pneumothorax.

There are studies investigating the (abnormalities) disorders of bone and cartilage metabolism in PE and PSP. However, no common reason was found for both of the diseases [3, 4]. Height measurements were higher in PSP patients compared to subjects in the control group, whereas BMI and weight measurements were found to be lower. Bone mineral density measurements were lower than the control group. These differences were statistically significant. 25-OH vitamin D levels were found significantly lower in PSP patients [3].

Accordingly, biomechanical, histopathological, and morphological properties of sternum and costal cartilage were investigated in patients with PE. Biomechanical stability was found to be decreased in these patients, and this result was attributed to collagen disorder [4].

Besides, genetic studies could not reveal the pathogenesis of both PE and PSP. Gpr126 deletion was suggested to be a common genetic factor in patients with PE and scoliosis [5].

The etiology of PE has not been fully elucidated yet. It has been shown to be associated with systemic connective tissue diseases. About 66% of the patients with Marfan syndrome also have pectus deformities [9]. Another study in patients with Marfan syndrome has shown that patients with primary spontaneous pneumothorax and Marfan syndrome have a flatter chest structure than the control group [10]. There is also a familial predisposition. Approximately half of the patients have relatives with skeletal changes. However, a direct genetic link has not been shown [11].

Pectus excavatum is a deformity that can be treated with two different surgical techniques. The Ravitch procedure (1949) is an open surgery technique that has been used for many years but since 1998, a minimally invasive Nuss technique has been performed more frequently [9]. Surgical repair is mostly done with cosmetic indications. A significant proportion of the patients has no symptoms [11].

There are a number of hypotheses related to PE etiology. Some of these are intrauterine compression, pulmonary restriction, diaphragm changes, osteogenesis, and inadequate chondrogenesis. An important portion of the studies has focused on the histology and biochemistry of the costal cartilages. In these studies, the metabolism and composition of type 2 collagen found in the costal cartilage have been investigated [11].

The primary spontaneous pneumothorax is more common in males, tall and thin people, and smokers. Exposure to atmospheric pressure changes is also one of the risk factors [12]. In a large study that investigated the risk factors for the development of pneumothorax in smokers with chronic obstructive pulmonary disease (COPD), the risk of pneumothorax was found to be higher in male gender, non-Hispanics, and patients with emphysema. The risk of pneumothorax was found to be independent of patients' lung functions and heights [12]. Prolonged air leak is one of the most common complications after spontaneous pneumothorax operations. Bullae diameter, advanced age, American Society of Anesthesiologists score, and bilateral procedures were risk factors for prolonged air leak [13].

Indications for surgery in PE are cosmetic and aesthetic complains in general. Majority of the patients have mild deformities and may not require any treatment. In this study, we examined the risk of developing spontaneous pneumothorax in this group of patients. In the literature, most of the studies that have investigated the relationship between PE and pneumothorax include pneumothorax that develops as a complication of the pectus operations [14, 15]. The relationship between chest configurations and the development of primary spontaneous pneumothorax was investigated in a recent study published by Park et al. In this study, it has been noted that patients with primary spontaneous pneumothorax had a different thoracic configuration. In the same study, it has also been showed that patients with anteroposteriorly flatter, laterally narrower, and craniocaudally taller thorax were more likely to develop pneumothorax [16].

## 5. Conclusion

Both diseases may have a common etiological factor, regarding polygenic character of the diseases with variable penetration. Further studies on larger series of patients are needed in order to reveal possible common etiological factors in PE and PSP. If it is the case, people who have chest deformities might be warned during childhood and adolescence to take precautions (smoking cessation, weight gain, exercise, etc.).

## Figures and Tables

**Table 1:** Exclusion criteria.

(i) Secondary pneumothorax
(ii) Additional diseases
(iii) Marfan syndrome or Marfan-like body structure
(iv) Relapse pneumothorax
(v) Recurrent pneumothorax
(vi) Patients under 20 years old or above 35 years old

**Table 2:** Diagnoses of the patients in the control group (group II).

Number	Gender	Diagnosis
1	M	Thorax trauma
2	M	Rib fracture
3	M	Pneumonia
4	M	Traffic accident
5	M	Hemoptysis
6	M	Pneumonia
7	M	Nodule in lung
8	M	Gunshot
9	M	Stabbing
10	F	Rib fracture
11	M	Nodule in lung
12	M	Rib fracture
13	M	Rib fracture
14	M	Hydatid cyst
15	M	Traffic accident
16	M	Stomach tumor
17	F	Rib fracture
18	M	Diaphragm elevation
19	M	Thymic hyperplasia
20	F	Gunshot

## Data Availability

The datasets used and/or analysed during the current study are available from the corresponding author upon reasonable request.

## Abbreviations

PE: Pectus excavatum
COPD: Chronic obstructive pulmonary disease.
